# On the complexity of helical tomotherapy treatment plans

**DOI:** 10.1002/acm2.12895

**Published:** 2020-05-04

**Authors:** Tania Santos, Tiago Ventura, Josefina Mateus, Miguel Capela, Maria do Carmo Lopes

**Affiliations:** ^1^ Physics Department University of Coimbra Coimbra Portugal; ^2^ Medical Physics Department IPOCFG, E.P.E Coimbra Portugal

**Keywords:** helical tomotherapy, plan complexity, principal component analysis, plan complexity score

## Abstract

**Purpose:**

Multiple metrics are proposed to characterize and compare the complexity of helical tomotherapy (HT) plans created for different treatment sites.

**Methods:**

A cohort composed of 208 HT plans from head and neck (105), prostate (51) and brain (52) tumor sites was considered. For each plan, 14 complexity metrics were calculated. Those metrics evaluate the percentage of leaves with small opening times or approaching the projection duration, the percentage of closed leaves, the amount of tongue‐and‐groove effect, and the overall modulation of the planned sinogram. To enable data visualization, an approach based on principal component analysis was followed to reduce the dataset dimensionality. This allowed the calculation of a global plan complexity score. The correlation between plan complexity and pretreatment verification results using the Spearman’s rank correlation coefficients was investigated.

**Results:**

According to the global score, the most complex plans were the head and neck tumor cases, followed by the prostate and brain lesions irradiated with stereotactic technique. For almost all individual metrics, head and neck plans confirmed to be the plans with the highest complexity. Nevertheless, prostate cases had the highest percentage of leaves with an opening time approaching the projection duration, whereas the stereotactic brain plans had the highest percentage of closed leaves per projection. Significant correlations between some of the metrics and the pretreatment verification results were identified for the stereotactic brain group.

**Conclusions:**

The proposed metrics and the global score demonstrated to be useful to characterize and quantify the complexity of HT plans of different treatment sites. The reported differences inter‐ and intra‐group may be valuable to guide the planning process aiming at reducing uncertainties and harmonize planning strategies.

## 1 INTRODUCTION

Intensity‐modulated radiation therapy (IMRT) is becoming a standard treatment technique for cancer patients, using either conventional linear accelerators (linac) or dedicated technologies such as helical tomotherapy (HT). In HT, the radiation beam produced by a compact linac is collimated to a fan‐beam. Delivery is done while the beam rotates around the patient and the couch translates through the gantry ring. Both the rotation and translation speeds are constant throughout the treatment. Modulation of beam intensity is accomplished by a pneumatically driven binary multileaf collimator (MLC). An arc‐shaped detector array, mounted opposite to the linac, records the exit radiation signal, which can be used for patient positioning verification, plan deliverability evaluation or machine quality assurance (QA).^1^

For treatment planning, parameters like the field width, the pitch and the initial modulation factor are manually set, while each MLC leaf open time per projection (51 by gantry rotation) is determined during the optimization phase. As many authors have reported,^2^, ^3^, ^4^, ^5^ a suboptimal choice of these parameters can compromise plan quality and deliverability, as well as increase treatment time. Therefore, several optimal values and planning approaches have been suggested. For instance, Kissick et al.^4^ proposed a rule to choose the pitch values and minimize the longitudinal ripple effect — thread effect — characteristic of HT plans. Shimizu et al.^6^ presented a method to derive an initial modulation factor and a site‐specific upper limit for this parameter to reduce the delivery time without compromising plan quality. Westerly et al.,^2^ using a subset of plans with unexpectedly poor pretreatment QA results, found that these plans had a high percentage of small leaf open times (LOT), the mean LOT being <100 ms. After replanning, the mean LOT became higher than 100 ms and the deviations between calculated and measured dose fell within ±3%. This could have happened due to the inaccuracies associated with the modeling of the MLC leaf latency in the treatment planning system (TPS) whose impact is higher for short leaf open times. Multileaf collimator leaf latency and tongue‐and‐groove/penumbra effects have indeed been pointed as factors that can affect plan deliverability.^2^, ^7^

More comprehensive studies for different treatment sites, including a wider set of TPS reported parameters, such as the couch travel, couch speed, number of gantry rotations, gantry period and treatment time, have been carried out.^8^, ^9^ Bresciani et al.,^8^ using 384 HT plans of multiple treatment sites, found no strong correlations between some of these factors and the results of pretreatment QA verification. Binny et al.^9^ have used multiple statistical process control methods on a set of head and neck (28), pelvic (19) and brain (23) plans, to define lower and upper limits for planning parameters, like the modulation factor, gantry period, and couch speed, based on acceptable pretreatment QA results. The established ranges were specific to each treatment site and contributed to improve the treatment efficiency at their institution.

Given the numerous degrees of freedom existing in HT, plans created for the same site may have different degrees of complexity, which may not be fully characterized by the TPS reported parameters. The evaluation of radiotherapy plans complexity has been widely researched. Multiple metrics have been proposed for static‐gantry IMRT and volumetric modulated arc therapy.^10^, ^11^, ^12^, ^13^, ^14^, ^15^ Complexity analysis has demonstrated to play a role in treatment plans characterization and comparison, contributing to adapt and improve the planning, optimization and QA processes. To date, a comprehensive evaluation of the helical tomotherapy plans complexity, through the definition and extension of some existing metrics is lacking in the literature. Thus, this study aims to quantify, evaluate and compare the complexity of HT plans created for various treatment sites by calculating several metrics. These metrics include some commonly evaluated parameters and novel indices that assess different aspects of the HT plans which may directly or indirectly contribute to increased uncertainties in dose calculation and delivery. The potential effect of complexity on the plans deliverability was also investigated.

## 2 MATERIALS AND METHODS

### 2.A Treatment plans and deliverability evaluation

A total of 208 plans from patients who underwent helical IMRT treatments at our institution were retrospectively analyzed. The considered treatment sites included head and neck (105), prostate (51) and brain tumor cases (52). The head and neck plans were generated with simultaneously integrated boost, for two or three dose levels. The prescription dose per fraction to the high‐risk planning target volume (PTV) was 2 or 2.12 Gy. In prostate tumor cases, only plans aiming to irradiate the prostate and seminal vesicles or the involved fossa, with a dose per fraction ranging from 2 to 2.5 Gy were selected. Metastatic brain tumors were irradiated with stereotactic radiosurgery, with the prescription doses varying between 19 and 22 Gy in a single fraction.

All plans were created in the Tomotherapy treatment planning system v.5.1.1.6 (Accuray Inc., Sunnyvale, CA, USA) to be delivered by a Tomotherapy HD unit (Accuray Inc., Sunnyvale, CA, USA). A field width of 2.5 cm in dynamic jaw mode^16^ was considered for head and neck and prostate cases and 1 cm for stereotactic brain plans. The initial modulation factor was set according to the planner’s preferences and the adopted pitch values were based on published guidelines.^4^, ^5^

To evaluate plan deliverability, that is, the agreement between planned and measured dose, pretreatment QA verification results were retrospectively collected. All plans had been recalculated in the Tomotherapy phantom (Cheese phantom) and delivered with the couch out of the bore. Dosimetry Check software v.5.5 (LifeLine Software Inc., Austin, TX, USA) was used to reconstruct the measured dose distribution from the acquired sinogram ^17^, ^18^. Three‐dimensional global gamma analysis was performed with 3% of maximum dose/3 mm distance‐to‐agreement criteria and 10% dose threshold (TH) for head and neck and prostate, and 3%/2 mm 10%TH for stereotactic brain plans. The passing rate acceptance limit was 95%. For the purpose of this work, more stringent criteria were also adopted, namely 3%/2 mm 10%TH for head and neck and prostate and 2%/2 mm 10%TH for stereotactic brain cases. For stereotactic brain plans, a Gafchromic EBT3 film (Ashland Inc., Covington, Kentucky, USA) was also used to assess the dose distribution in a coronal plane of the Cheese phantom. Films were scanned in a flatbed scanner Epson Expression 10000 XL (Seiko Epson Corporation, Japan) and a home‐made software was utilized for film processing, applying triple‐channel dosimetry.^19^ Global gamma analysis was performed with a criterion of 3%/2 mm, in a dedicated Tomotherapy station. The passing rate acceptance limit was again 95%. Point dose measurements were also performed using an Exradin A1SL chamber (Standard Imaging, Middleton, WI, USA) placed in the same phantom at the center of the emulated brain lesion. A difference between the planned and measured dose of ±3% was considered acceptable.

### 2.B Complexity metrics

For each plan, 14 complexity metrics, including some commonly evaluated parameters and novel metrics, have been computed from the planned sinogram using an in‐house program developed in MATLAB R2017b (Mathworks, Natick, MA, USA). The planned sinogram is a two‐dimensional matrix with information on the fraction of time that a given MLC leaf is open per projection, relative to its duration and it is saved in the DICOM RT plan.

The computed parameters included the actual modulation factor (MF). The MF is defined as the ratio between the maximum leaf open time and the mean of all nonzero leaf opening times. A higher MF allows a larger range of beamlet intensities.^6^ The mean LOT (in ms), the percentage of open leaves with an opening time below 100 ms (%LOT < 100 ms), 50 ms (%LOT < 50 ms), 30 ms (%LOT < 30 ms), and the percentage of leaves with an opening time close to the projection duration (%LOT > pT‐20 ms) were also calculated to characterize the leaf open times distribution. As reported^2^, ^5^, ^7^ the linear model assumed for the MLC leaf latency is violated for small leaf open times and LOTs approaching the projection duration, which may compromise plan deliverability. Therefore, plans with a lower mean LOT and a higher percentage of leaves with a short LOT and/or approaching the projection time were considered more complex.

The treatment time divided by the prescribed dose per fraction — TT/Gy — is here presented as a simple indicator of complexity. It depends somehow on the longitudinal extension of the target volume and on the pitch. Plans with a higher TT/Gy may be considered more complex.

To evaluate the modulation of the entire planned sinogram, that is, the differences in leaf open times, three metrics are proposed. These are extensions to HT of indices previously defined for conventional IMRT techniques. The leaf open time variability (LOTV), adapted from,^12^ is calculated for each leaf that opens at least once during the treatment as:

(1) $${\text{LOTV}}_{\text{leaf}}=\frac{\sum_{k=1}^{N_{\text{CP}}-2}t_{\max}-\left|f\text{LOT}\left(k,\text{leaf}\right)-f\text{LOT}\left(k+1,\text{leaf}\right)\right|}{\left(N_{\text{CP}}-2\right)\times t_{\max}}$$

where $t_{\max}$ is the maximum LOT for that leaf across all control points (CP), fLOT is the matrix corresponding to the planned sinogram and $N_{\text{CP}}$ the total number of control points that is equal to the number of projections + 1. The plan LOTV corresponds to the average over all leaves. This index ranges from 0 to 1, being 1 when all leaves have the same opening time. The higher the LOTV, the lower the variations in leaf open times along the treatment, and therefore, the lower the plan modulation.

The Plan Time Sinogram Variation (PSTV), an adaptation of the plan intensity map modulation score proposed by Coselmon et al.,^11^ is computed for a given control point by summing the LOT differences in two directions:

(2) $${\text{PSTV}}_{\mathrm{CP}}=\sum_{j=1}^{N_{l}-1}\left|fLOT\left(CP,j\right)-fLOT\left(CP,j+1\right)\right|+\left|fLOT\left(CP,j\right)-fLOT\left(CP+1,j\right)\right|$$

where $N_{l}$ is the total number of MLC leaves (64). The PSTV for the plan is calculated as the mean of all $PSTV_{\mathrm{CP}}$ , as all the other indices defined hereafter. The larger the PSTV score the higher the MLC leaves open time variation, and therefore the plan modulation.

The Modulation Index (MI)^10^ was here modified to quantify the leaf open time variations in the planned sinogram in the directions defined by the MLC leaves $\left(x\right)$ , the projections/control points $\left(y\right)$ and the corresponding diagonals $\left(xy,yx\right)$ . The number of LOT changes (Δt) between adjacent elements in the four directions that exceed a certain fraction ( f ) of the standard deviation ( σ ) of the entire planned sinogram was calculated $N\left(f;\Delta t>f\sigma \right)$ and hence the mean per projection:

(3.1) $$Z_{x}\left(f\right)=\frac{1}{N_{\mathrm{CP}}-1}N_{x}\left(f;\Delta t_{x}>f\sigma \right)$$

(3.2) $$Z_{y}\left(f\right)=\frac{1}{N_{\mathrm{CP}}-1}N_{y}\left(f;\Delta t_{y}>f\sigma \right)$$

(3.3) $$Z_{\mathrm{xy}}\left(f\right)=\frac{1}{N_{\mathrm{CP}}-1}N_{\mathrm{xy}}\left(f;\Delta t_{\mathrm{xy}}>f\sigma \right)$$

(3.4) $$Z_{\mathrm{yx}}\left(f\right)=\frac{1}{N_{\mathrm{CP}}-1}N_{\mathrm{yx}}\left(f;\Delta t_{\mathrm{yx}}>f\sigma \right)$$

where the total number of projections is equal to $N_{\mathrm{CP}}-1$ and f=0.01:0.01:2 ^.^ ^10^ The total $Z\left(f\right)$ represents the spectrum of such changes in the entire sinogram and it is given by:

(4) $$Z\left(f\right)=\frac{Z_{x}\left(f\right)+Z_{y}\left(f\right)+Z_{\mathrm{xy}}\left(f\right)+Z_{\mathrm{yx}}\left(f\right)}{4}$$

The modulation index corresponds to the area under the spectrum:

(5) $$MI=\int_{0}^{2\sigma }Z\left(f\right)\;df$$

The larger the value of MI, the higher the plan modulation ^10^.

To assess the amount of tongue‐and‐groove effect in HT, two novel metrics are defined. The leaves with zero open neighbors score (L0NS) and the leaves with one open neighbor score (L1NS) are calculated for a given control point as:

(6) $${\text{L0NS}}_{\text{CP}\;}\left(\%\right)=\frac{{\text{Number of open leaves with zero open neighbors}}_{\text{CP}}}{{\text{Number of open leaves}}_{\text{CP}}}\times 100$$

(7) $${\text{L1NS}}_{\text{CP}}\left(\%\right)=\frac{{\text{Number of open leaves with one open neighbor}}_{\text{CP}}}{{\text{Number of open leaves}}_{\text{CP}}}\times 100$$

Due to the tongue‐and‐groove/penumbra blur effects, the primary fluence under a given MLC leaf varies according to the state of its neighbors. Such differences are taken into account in the TPS during the end‐of‐planning process, through a leaf‐by‐leaf basis correction.^2^, ^20^ The presented indices quantify the number of times that those corrections need to be applied, and eventually their accuracy.

The closed leaf score (CLS), adapted from,^13^ is computed per control point as the ratio of closed leaves to all MLC leaves (64):

(8) $$CLS_{\mathrm{CP}}\left(\%\right)=\frac{\sum_{j=1}^{N_{l}}\left(fLOT\left(CP,j\right)=0\right)}{N_{l}}\times 100$$

The CLS can vary between 0 and 100%, being 100% when all leaves are closed during the treatment. This index is partially related to the target volume. However, when the number of closed leaves per CP is high, it is assumed that a plan can be considered more complex due to the possible significant impact of mechanical errors and dose calculation uncertainties.

The percentage of closed leaves within the so‐called treatment area, defined by the right most and left most open leaves in a given control point was also calculated (CLS_in_). It gives an indication of the complexity of the irradiation pattern, due to the target volume irregularity and/or its proximity with critical structures. Thus, the higher the CLS_in_ the greater the plan complexity.

### 2.C Statistical analysis

Statistical analysis was performed in MATLAB. The mean and standard deviation of the 14 complexity metrics were calculated for the three groups of plans. As each plan is characterized by a vector of 14 features, as many as the considered complexity metrics, it is difficult to summarize, visualize and identify patterns in the data, namely differences between the groups. To reduce the dimensionality of the dataset and enable graphical epresentation of the distribution of plans, principal component analysis (PCA) was performed.^21^ Principal component analysis is a multivariate technique widely used in dataset dimensionality reduction to increase interpretability while preserving most of the initial information.^22^, ^23^, ^24^. For that, PCA finds a new set of uncorrelated variables (principal components, PCs) that result from linear combinations of the original ones and that successively maximize variance. The weight of each variable for every PC is known as its loading. The resulting number of PCs is equal to the number of original variables. To decide how many PCs to retain, a common approach consists in defining a cut‐off (70%–90%) of the cumulative percentage of the total variance explained and considering the minimum number of PCs that exceed that cut‐off. Another possibility consists in representing by the so‐called scree plot, the variance associated with each PC vs PC number and base the decision on the analysis of the slope change between adjacent line segments.^21^

In this study, all the 14 complexity metrics were considered for PCA analysis that corresponded to a data matrix X with 14 metrics (columns) for the 208 plans (rows). Those metrics had different units of measurement and numerical ranges, which affects the variance. To ensure that all variables would contribute equally to the analysis, data were standardized before performing PCA, such that all metrics had a mean of 0 and variance of 1.^25^ The PCA analysis output consisted of 14 principal components. To determine the number of PCs to keep for data representation, a cut‐off of 70% of the total variance explained was adopted.^21^ The scree plot for the HT complexity data is given in Figure S1.

Still using PCA, after modifying some metrics such that all increased with increasing complexity, the methodology proposed by the authors in a previous work^25^ was followed to compute a global plan complexity score (PCS). This score aims at characterizing and comparing the treatment plans through a single indicator and it is calculated as the weighted mean of the selected principal components:

(9) $$\text{Plan Complexity Score}=\sum_{l=1}^{L}\frac{v_{l}}{v}\times PC_{l}$$

where L is the minimum number of PCs corresponding to a cumulative percentage of the total variance explained higher than 70%, v is the total variance explained by the retained PCs and $v_{l}$ the percentage of variance explained by $PC_{l}$ .

The absolute value of the PCS may not be easy to interpret, as explained in Santos et al.^25^ Therefore, a normalized version of this score, nPCS , was calculated for a given plan i within the set of plans as:

(10) $$nPCS_{i}=\frac{PCS_{i}-\min\;PCS}{\max\;PCS-\min\;PCS}$$

nPCS is 0 for the plan with the minimum PCS (min PCS) and 1 for the plan with the maximum PCS (max PCS). The higher the value of nPCS, the greater the plan complexity for the set of plans considered in the study.

The correlation between pretreatment QA verification results, obtained for each group of plans, computed complexity metrics, and nPCS values was investigated using Spearman’s rank correlation coefficients, for a significance level of 5%. Depending on the absolute value of r_s_, the dependency was classified as: 0–0.19 “very weak”, 0.20–0.39 “weak”, 0.40–0.59 “moderate”, 0.60‐–0.79 “strong” and 0.80–1 “very strong”.^26^

## 3 RESULTS

### 3.A Treatment plans

Some of the TPS reported parameters for the considered groups of plans (head and neck, prostate and stereotactic brain) are summarized in Table 1. It can be seen that stereotactic brain plans have the longest gantry period and the highest number of gantry rotations, as well as the smallest pitch (0.100 for all plans), couch speed and couch travel. This is expected due to the high dose delivered in a single fraction (19‐22 Gy) and the small target volume (5.9 ± 5.1 cc, on average). Head and neck cases, on the other hand, present the highest pitch, fastest gantry period and couch speed. The couch travel gives an indication of the craniocaudal extension of the treatment region, being higher for the head and necks plans.

**Table 1:** Summary of some conventional parameters (mean ± standard deviation) for the three groups of HT plans.

	Head and neck	Prostate	Stereotactic brain
	(n = 105)	(n = 51)	(n = 52)
Pitch	0.408 ± 0.032	0.348 ± 0.042	0.100 ± 0.000
Gantry period (s)	16.4 ± 1.6	23.6 ± 3.4	46.9 ± 5.3
Gantry rotations	18.1 ± 2.4	12.2 ± 2.1	26.9 ± 7.1
Couch travel (cm)	18.5 ± 1.8	10.5 ± 1.6	2.8 ± 0.7
Couch speed (cm/s)	0.0629 ± 0.0068	0.0376 ± 0.0060	0.0023 ± 0.0002

### 3.B Complexity metrics

**Fig. 1:**
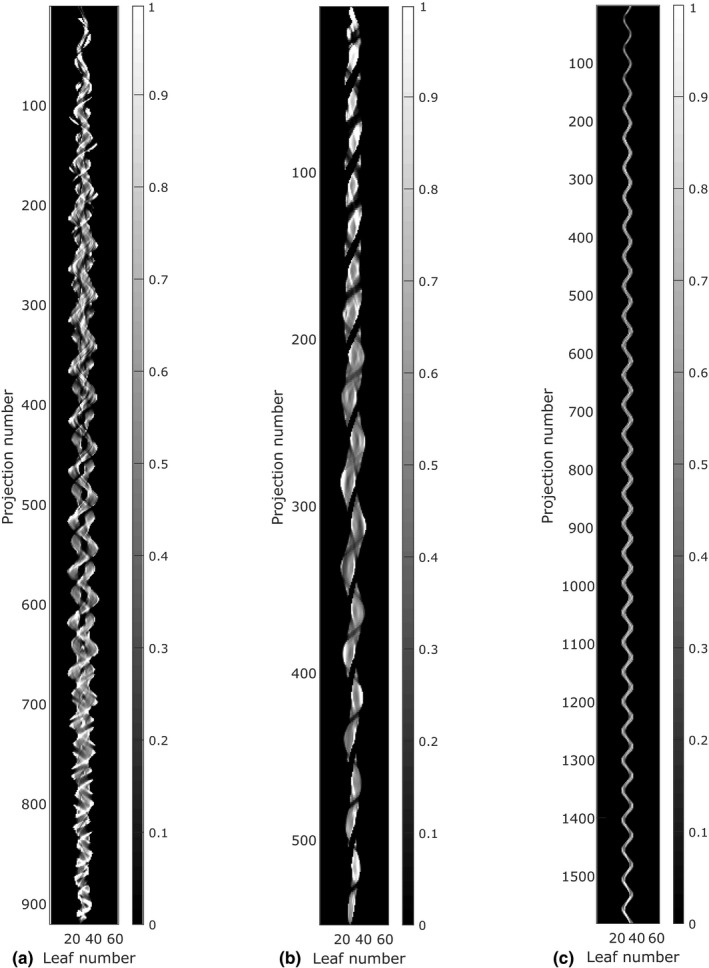
Planned sinograms for a typical head and neck (a), prostate (b) and stereotactic brain (c) case. Each image element (row, column) represents the fraction of time (0–1) that a given MLC leaf is open relative to the projection duration.

Plans from various treatment sites were included in this study to appreciate the differences in terms of complexity between them based on the analysis of the planned sinogram. Figure 1 displays a representative example of a planned sinogram for each group. As Fig. 1 illustrates, the head and neck plans typically have a more complex irradiation pattern. The prostate cases usually have a smaller number of projections whereas, the stereotactic brain plans are characterized by a low number of MLC open leaves per projection.

**Fig. 2:**
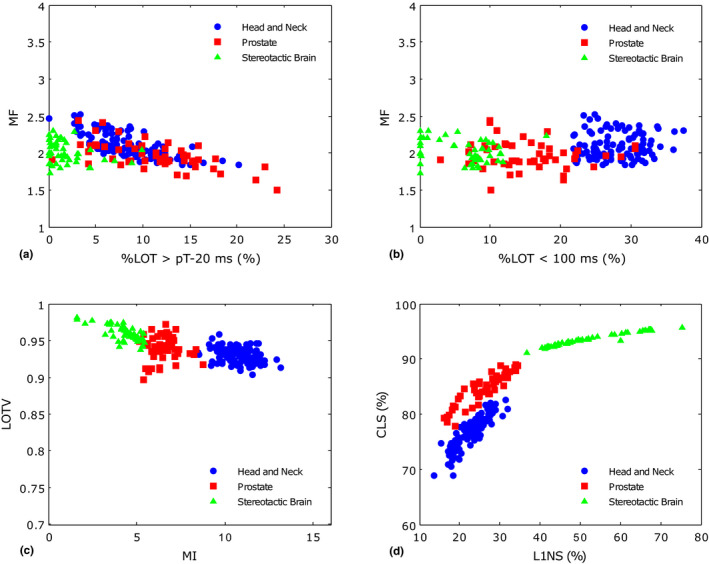
Representation of four pairs of complexity metrics for the three treatment sites.

The visual differences in the planned sinogram complexity were quantified through the calculation of several metrics. Four pairs of those metrics are presented in Fig. 2. Figure 2(a) shows that the percentage of leaves with an open time close to the projection time (%LOT > pT‐20 ms) tends to be higher for plans with a lower modulation factor (MF), especially when considering head and neck and prostate plans. Nevertheless, there are no obvious differences in the modulation factor between the groups. In Fig. 2(b) the percentage of leaves with an opening time below 100 ms (%LOT < 100 ms) is generally the highest in the head and neck plans and the lowest in stereotactic brain ones. No clear correlation was identified between the modulation factor within each group and the percentage of small LOT. Figure 2(c) indicates that the modulation index allows a clear distinction between the groups of plans. LOTV did not change much among the groups meaning that the individual leaf open time variations along the treatment are generally quite smooth. The closed leaf score (CLS) was, on average, higher for the stereotactic brain plans as could be anticipated due to the small volume of the metastatic lesions — Fig. 2(d). The percentage of leaves with one open neighbor evaluated by the L1NS is also the highest in this group, which is also somehow expected. As the number of open leaves per projection is reduced, the leaves in the extremities of the treatment area have more impact in its calculation.

A summary of the complexity metrics computed for the three groups of HT plans is presented in Table 2.

**Table 2:** Average of the complexity metrics (mean ± standard deviation) for the three groups of HT plans.

	Head and neck	Prostate	Stereotactic brain
	(n = 105)	(n = 51)	(n = 52)
Modulation factor (MF)	2.096 ± 0.173	1.966 ± 0.184	2.027 ± 0.151
TT/Gy (s/Gy)	141.6 ± 20.6	133.2 ± 28.5	60.1 ± 12.9
Mean LOT (ms)	153.2 ± 9.4	234.7 ± 26.7	452.7 ± 35.1
%LOT < 100 ms (%)	27.9 ± 3.6	15.5 ± 6.3	5.9 ± 4.5
%LOT < 50 ms (%)	9.7 ± 1.8	5.8 ± 2.7	2.9 ± 2.4
%LOT < 30 ms (%)	3.7 ± 0.8	2.3 ± 1.0	1.3 ± 1.1
%LOT> pT‐20 ms (%)	8.7 ± 3.7	11.2 ± 5.2	1.8 ± 2.2
LOTV	0.931 ± 0.010	0.939 ± 0.016	0.960 ± 0.010
PSTV	5.4 ± 0.7	3.4 ± 0.7	1.8 ± 0.5
Modulation index (MI)	10.7 ± 0.9	6.6 ± 0.8	4.3 ± 0.9
CLS (%)	76.7 ± 3.0	84.6 ± 2.9	93.4 ± 1.2
CLS_in_ (%)	10.3 ± 4.5	5.4 ± 4.0	0.2 ± 1.5
L0NS (%)	0.9 ± 0.5	1.4 ± 1.2	0.0 ± 0.1
L1NS (%)	22.8 ± 3.8	26.2 ± 5.2	51.7 ± 9.7

From the computed metrics, PCA was performed to reduce the dataset dimensionality. In PCA, the first two principal components together explained 76.5% of the total variance, 65.2% and 11.3%, respectively, which is above the predefined cut‐off (70%). Therefore, the resulting two‐dimensional representation of the data can be considered a good approximation of the original scatter plot in 14 dimensions.

**Fig. 3:**
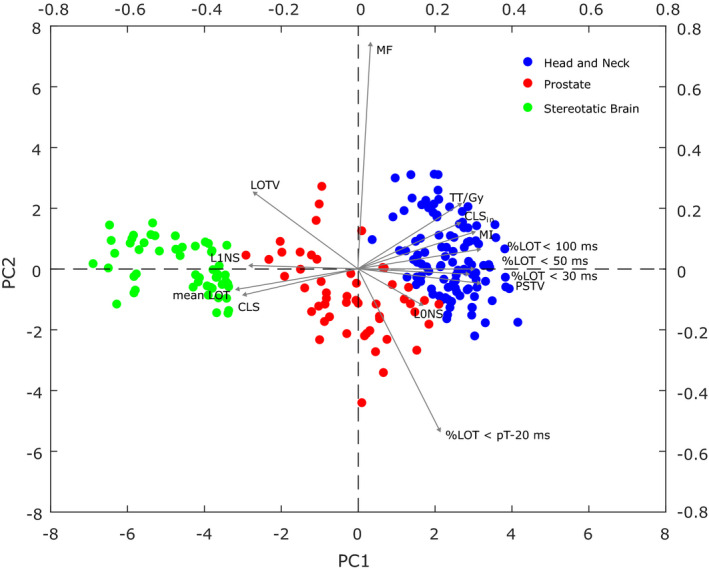
Biplot for the HT treatment plans data. Each data point corresponds to a plan and the weights of each variable are represented by a vector.

Figure 3 presents the PC2 vs PC1 for the HT data, where each point corresponds to a plan (primary axis). The metrics are also displayed, as vectors, where the x‐component represents the weight of that variable for the PC1 and y‐component the weight for the PC2 (secondary axis). This kind of representation provides information on the correlation between the 14 complexity metrics for the entire set of plans. If two variables have a large positive association, the corresponding vectors are close together; if it is weak, the angle between them is around 90° and when negative and strong the angle is close to 180°. For instance, metrics that assess the percentage of leaves with small leaf open times (%LOT < 100 ms, %LOT < 50 ms and %LOT < 30 ms) present a very strong dependency, r_s_ > 0.8. These indices are in turn, negatively correlated with the mean LOT, which indicates, as expected, that plans with a smaller mean LOT have a higher percentage of short leaf open times. None of these parameters is associated with the modulation factor (MF). The %LOT > pT‐20 ms, on the contrary, confirmed to be negatively correlated with the modulation factor. To further illustrate this inverse relationship, the TPS LOT histograms of three prostate cases are provided in the Fig. S2. Regarding the indices that evaluate the variations of leaf open times, the relationship between the modulation index (MI) and the LOTV is negative, r_s_ = −0.691, whereas it is positive with the PSTV. These indices (MI, LOTV and PSTV) are in turn, strongly associated with the CLS_in_ that evaluates the percentage of closed leaves within the treatment area. Finally, the L1NS presents a very strong relationship with the CLS_,_ r_s_ = 0.891, as referred above.

From the distribution of the data points, three clusters can be fairly identified, corresponding to the considered groups of plans, indicating that they have generally different levels of complexity. Further analysis of the relative position of the data points and the variables can explain groups' separation. PC1, represented by the horizontal axis, accounts for 65.2% of the total variation of the dataset and it is indeed the PC that more clearly separates the groups of plans. Plans on the left side of the graph, having large negative values for PC1, tend to have higher values for the variables with larger negative loadings for that PC and lower values for the variables with large positive loadings. Therefore, stereotactic brain plans, on the left side of the figure, must have the highest mean LOT, percentage of closed leaves per projection (CLS), L1NS and LOTV. Head and neck plans, on the right side, tend to have the highest percentage of small leaf open times (%LOT < 100 ms, %LOT < 50 ms and %LOT < 30 ms), MI, PSTV, and percentage of closed leaves within the treatment area (CLS_in_). These findings are corroborated by the information in Table 2. Prostate plans are mostly in between the other two groups, except some cases that are overlapped with the head and neck one. Looking now at the vertical axis, PC2, the modulation factor (MF), and the %LOT > pT‐20 ms are the variables that mostly contribute to this PC. But while plans on the top of the figure tend to have a higher modulation factor, those towards the bottom have a higher percentage of leaves with an open time close to the projection duration.

**Fig. 4:**
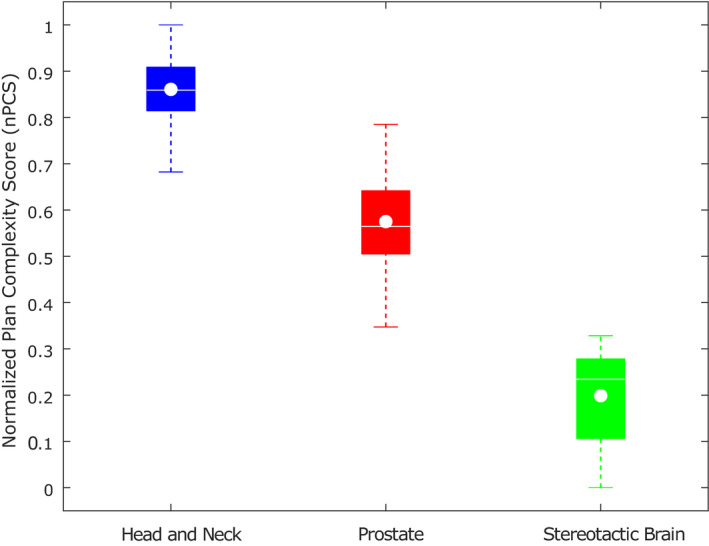
Boxplots summarizing the normalized plan complexity score (nPCS) obtained for the three groups of plans. On each box, the bottom and top edges indicate the 25th and 75th percentiles, respectively — interquartile range. The median value is represented by a white horizontal line and the mean value by a white “o”. The whiskers go down to the minimum and up to the maximum data values within 1.5 times the interquartile range.

To summarize the information provided in the biplot and compare the global complexity inter‐ and intra‐ group of plans, the normalized plan complexity score (nPCS) was calculated — Fig. 4. As the median line of each boxplot in Fig. 4 lies outside the other boxes, the groups of plans can be considered different in terms of global complexity. The head and neck plans are generally the most complex, followed by the prostate and the stereotactic brain ones. Stereotactic brain plans have the highest interquartile range whereas the prostate group presented the largest spread of nPCS, as indicated by the extreme values of the corresponding boxplot.

### 3.C Correlation between the complexity metrics and pretreatment QA results

Pretreatment verification results were acceptable for all plans, with no significant differences between the groups. The 3D global gamma passing rate for the head and neck cases was on average 98.6 ± 1.0% and 97.6 ± 1.9% for the prostate plans, considering a 3%/3 mm, 10%TH criteria. The average results for the stereotactic brain group were: 98.7 ± 2.5% — 3D global gamma passing rate (3%/2 mm, 10%TH), 0.1 ± 1.1% — IC percentage deviation, and 98.5 ± 1.5% — film gamma passing rate (3%/2 mm).

The correlation between each complexity metric/global complexity score and the results of pretreatment QA verification obtained for each group of plans was investigated through the calculation of the Spearman’s rank correlation coefficients. For the head and neck and prostate cases, no significant correlations have been identified, neither when considering each metric individually nor the nPCS (Table S1). For the stereotactic brain group, the obtained Spearman’s rank correlation coefficients and corresponding p‐values are presented in Table 3. Some moderate and strong dependencies have been obtained for this group of plans. The correlations tended to be stronger when more stringent analysis criteria were adopted for 3D global gamma analysis. Nevertheless, ionization chamber results were not related with any of the computed metrics.

**Table 3:** Spearman’s correlation coefficients, r_s_, and corresponding p‐values (within brackets) between 3D gamma passing rates with various criteria, ionization chamber percent difference (IC %diff), film results and the complexity metrics/nPCS for the stereotactic brain plans. Correlations were considered statistically significant for a *P* < 0.05. Values in bold correspond to significant moderate or strong correlations.

	3D global gamma analysis		IC %diff	Film 3%/2 mm
	3%/2 mm 10%TH	2%/2 mm 10%TH		
Modulation factor	0.148 (0.294)	0.320 (0.021)	−0.076 (0.594)	0.385 (0.008)
TT/Gy	−0.346 (0.012)	**−0.659 (0.000)**	−0.185 (0.189)	**−0.463 (0.001)**
Mean LOT	−0.035 (0.804)	0.327 (0.018)	0.138 (0.330)	0.203 (0.176)
%LOT < 100 ms	−0.144 (0.307)	−0.373 (0.006)	0.056 (0.692)	−0.329 (0.025)
%LOT < 50 ms	−0.168 (0.233)	−0.353 (0.010)	0.080 (0.575)	−0.363 (0.013)
%LOT < 30 ms	−0.215 (0.127)	−0.387 (0.005)	0.119 (0.399)	−0.393 (0.007)
%LOT> pT‐20 ms	−0.303 (0.029)	**−0.463 (0.001)**	−0.082 (0.565)	**−0.440 (0.002)**
LOTV	0.278 (0.046)	0.349 (0.012)	−0.041 (0.772)	0.303 (0.040)
PSTV	−0.291 (0.036)	**−0.436 (0.001)**	0.111 (0.432)	−0.337 (0.022)
Modulation index (MI)	−0.231 (0.100)	−0.329 (0.018)	0.091 (0.522)	−0.023 (0.878)
CLS	0.378 (0.006)	**0.655 (0.000)**	0.103 (0.466)	0.353 (0.016)
CLS_in_	−0.011 (0.937)	0.030 (0.832)	−0.039 (0.786)	0.018 (0.908)
L0NS	0.029 (0.839)	0.041 (0.775)	−0.009 (0.952)	−0.064 (0.671)
L1NS	0.395 (0.004)	**0.678 (0.000)**	0.089 (0.532	0.329 (0.025)
nPCS	−0.253 (0.070)	**−0.434 (0.001)**	0.024 (0.867)	**−0.442 (0.002)**

Plans with a higher total treatment time per Gy (TT/Gy) were significantly associated with poorer verification results, such that *r*_s_ = −0.463, *P* = 0.001 for film. The negative relationships observed for %LOT > pT‐20 ms also suggest that a high percentage of leaves with an opening time close to the projection duration may compromise plans deliverability. As for the metrics proposed to evaluate the variation in leaf open times, a significant dependency was observed between the PSTV and the 3D global gamma passing rates, *r*_s_ = −0.436, *P* = 0.001. The positive and strong dependency for the L1NS index and the more stringent 3D gamma analysis may indicate that the amount of tongue‐and‐groove does not have any influence on treatment deliverability. The same rationale applies to the CLS index that evaluates the percentage of leaves closed per control point. Lastly, the normalized plan complexity score showed a moderate association with the verification results (*r*_s_ > 0.4, *P* = 0.000).

The correlation between some of the TPS reported plan parameters, namely, the pitch, the gantry period, the number of gantry rotations, the couch travel and the couch speed and the pretreatment QA results was also investigated (Table S2). But once again, no significant dependencies have been identified for the head and neck and the prostate groups. Nevertheless for the stereotactic brain plans, all parameters, except pitch (0.100 for all plans) were significantly associated with the film gamma passing rates (*r*_s_ > 0.4, *P* < 0.05). The correlation was negative with the number of gantry rotations, couch speed, and couch travel and positive with the gantry period.

## 4 DISCUSSION

In this study, the complexity of a set of helical tomotherapy plans from head and neck, prostate and brain treatment sites was characterized and compared. To our knowledge, this is the first work that attempts to comprehensively extend the current discussion on plan complexity to HT, through both the definition of individual metrics and the calculation of a global complexity score.

In total, 14 complexity metrics were computed, including some commonly evaluated parameters and novel indices with the aim of assessing different features of the treatment plans. Due to the high number of metrics, an approach based on principal component analysis was followed to explore the correlation between them to find a subset of the most representative components. The visual representation of the data in two‐dimensions allowed the identification of similarities and differences between the considered treatment sites.

The head and neck plans were found to be the most complex for almost all the computed complexity indicators. Plans from this group had, for instance, the highest percentage of leaves with short opening times and the lowest mean LOT. Among the considered groups, the head and neck plans had the fastest gantry period which confirms the known influence of this parameter in the amount of leaves with small LOTs.^2^, ^27^ The score that evaluates the percentage of closed leaves within the treatment area (CLS_in_) indicated that the head and neck plans had also the most complex irradiation patterns which was confirmed by the calculated modulation indices, LOTV, PSTV and MI. The prostate plans had the highest percentage of leaves with an opening time approaching the projection duration (%LOT > pT‐20 ms). The %LOT > pT‐20 ms has shown to be inversely correlated with the modulation factor, which is in accordance with the findings by Binny et al.^27^ and Sevillano et al.^7^ The stereotactic brain plans had the highest percentage of closed leaves per projection, with just about five open leaves, on average, as well as the largest L1NS, which quantifies the amount of tongue‐and‐groove effect.

To summarize the information given by all complexity metrics, a global plan complexity score (nPCS) was calculated, following the methodology proposed by Santos et al.^25^ in the context of a national IMRT audit.^28^ The nPCS combines the multiple metrics into a single numerical score, allowing for the comparison of the relative complexity of the entire set of plans. Based on the nPCS values, the head and neck plans confirmed to be the most complex, followed by the prostate and the stereotactic brain ones. A higher complexity variability was observed for the prostate cases, presenting the larger range of nPCS values. This can be explained, in part, by the inclusion of patients with femoral prosthesis, demanding an adaptation of the typical planning strategy to reduce dose calculation uncertainties. The number of planners was also higher for the prostate group (6) than for the head and neck (3) and stereotactic brain (2) groups, being the adopted planning strategies largely dependent on the planners' skills and approaches.^29^ Yet, the reported differences in plan complexity intra‐ and inter‐group may also be due to factors such as variations in patient anatomy, PTV shape and/or volume, and in the dose constraints that may differ from one clinician to another. Plans complexity is also partially determined by the optimization algorithm that works like a black box. In HT, only the initial modulation factor, pitch and field width are set, and the actual modulation factor, number of projections and leaf open times result from the optimization process.^9^

The plans deliverability was weakly correlated with both the computed complexity metrics and the usual plan parameters for head and neck and prostate groups. Regarding the stereotactic brain plans, some moderate dependencies have been identified. Stereotactic brain plans with a higher total treatment time per Gy (TT/Gy) and/or with a higher percentage of leaves with opening times approaching the projection duration (%LOT > pT‐20 ms) were significantly associated with a poorer agreement (although within tolerance) between planned and measured dose. Plan parameters like the couch speed and the number of gantry rotations were also inversely correlated with the pretreatment QA results. These findings may be useful to establish clinical guidelines for planning of stereotactic brain cases at our institution. Accordingly, planners should carefully evaluate the TPS LOT distribution during the planning phase and aim to achieve a %LOT > pT‐20 ms less than the reported mean. The TT/Gy can also be easily assessed and it should be compared with the values obtained for similar clinical cases belonging to this group. The same rationale applies to the planning parameters.

All the 208 plans considered in this study had pretreatment QA results within the established tolerances, which is perhaps the main reason for the reported lack of significant correlations, mainly for the head and neck and prostate groups. A pool of plans with poorer verification results would perhaps allow for a clearer insight into the relationship between complexity and deliverability. Still, there are some factors that can mask the effect of complexity on delivery verification results. The first one is related to the pretreatment QA methods used. For the head and neck and prostate cases, the verification was based on 3D dose reconstruction using the signal recorded by the exit detector during delivery with the couch out of the bore. This methodology even having been deeply tested against film and ionization chamber measurements,^18^ may raise some questions related with the detector resolution, the dose calculation algorithm used (pencil beam) and even the sensitivity of the global gamma analysis.^30^, ^31^ TPS dose calculation (beam modeling, MLC modeling, commissioning inaccuracies) and MLC performance are also some potential sources of errors.

The impact of HT plan parameters on the pretreatment QA results has already been investigated by other authors.^2^, ^8^, ^9^ Westerly et al.,^2^ for instance, found that plans with a mean LOT below 100 ms, that is, using predominantly small leaf open times, were more likely to present significant deviations (>3%) between the calculated and the measured doses, in an analysis of six plans treated in a Tomotherapy Hi‐Art II system. Bresciani et al.^8^ have taken a pool of 384 HT plans to investigate if there were any treatment sites and/or plan parameters more likely to lead to poorer verification results. A wide variety of treatment sites were included, such as gynecological, head and neck, breast, lung, rectum, but no significant differences were noticed. As for the plan parameters, the correlations were rather weak. Binny et al.^9^ in a retrospective study with 28 head and neck, 19 pelvic and 23 brain HT plans, found no correlations between the TPS reported parameters, such as the gantry period and the percentage of leaf open times <100 ms, and the pretreatment QA results.

The characterization of HT plans complexity is particularly challenging. Due to the system particularities, defining what is more or less complex and which features to assess is not trivial. As most parameters reported by the TPS are intimately linked with each other and depend on the target volume and dose per fraction, it was decided not to include them in this study as complexity metrics. Also, new indices were defined that evaluate different aspects of the plans that may directly or indirectly contribute to increased uncertainties in dose calculation and delivery. These indicators demonstrated to be effective in quantifying the complexity of the plans. The reported differences inter‐ and intra‐ group suggest that it may be appropriate to define site‐specific recommendations to guide the planning and the QA processes. The values of both planning parameters and complexity metrics may be generally adopted as reference levels at our institution, as the pretreatment QA results of the plans included in this study were all clinically acceptable.

Some of the proposed complexity metrics, namely the modulation factor, TT/Gy, mean LOT, %LOT < 100 ms, %LOT < 50 ms, %LOT < 30 ms and the %LOT > pT‐20 ms can be directly assessed during the planning phase. Their comparison against local reference values may be used as a guide for planning and eventually contribute to harmonize local planning strategies. Planners should, along this line, carefully evaluate the percentage of leaves with a LOT approaching the projection duration and keep it as close as possible to the reference value for each site. Namely, in prostate cases where some atypically high values (>20%) have been identified and in stereotactic brain plans where a significant correlation with the pretreatment QA results has been noticed. The indices that evaluate the plan sinogram modulation and the amount of tongue‐and‐groove effect, on the contrary, can only be calculated after planning. Their calculation would not necessarily increase the time allocated to the QA process. Again, the comparison of the obtained values for a new plan with the reference ones may be useful to flag plans with a complexity higher than usual, which would be subject to a more rigorous QA.

## 5 CONCLUSIONS

In this study, the complexity of HT plans from different treatment sites was characterized and compared through the calculation of a set of metrics that evaluate multiple features of the planned sinograms. A statistical approach based on principal component analysis was followed to simplify data interpretation, allowing to explore the correlations among the proposed indices and quantify the differences in complexity between the studied groups of plans. Generally, head and neck plans were found to be the most complex for almost all metrics, which was confirmed by the computed global plan complexity score. The prostate plans had the highest complexity variability, which can be a result of a wider range of planning approaches.

The presented characterization of the differences inter‐ and intra‐ group of treatment sites may be useful to guide the treatment planning and the QA processes eventually reducing uncertainties and harmonizing local planning strategies.

## CONFLICT OF INTEREST

None.

## Supporting information


**Fig S1** **.** Scree plot of the principal components for the HT treatment plans data. The eigenvalues give the variance explained by each PC.

**Fig S2** **. **TPS leaf open time histogram for three similar prostate cases. Plans were created to irradiate the prostate and seminal vesicles with 2 Gy per fraction. (a) MF of 2.445 and %LOT > pT‐20 ms = 3.2%; (b) MF of 2.007 and %LOT > pT‐20 ms = 11.4%; (c) MF of 1.809 and %LOT > pT‐20 ms = 23.0%.

**Table S1** **. **Spearman’s correlation coefficients, r_s_, and corresponding p‐values (within brackets) between 3D gamma passing rates with various criteria and the complexity metrics/nPCS for the head and neck and prostate plans.

**Table S2** **. **Spearman’s correlation coefficients, r_s_, and corresponding *P* (within brackets) TPS reported parameters and the pre‐treatment QA results for the head and neck, prostate and SRS plans. Values in bold correspond to significant moderate or strong correlations.
