# Supplementary material for: On the complexity of helical tomotherapy treatment plans
Source: J Appl Clin Med Phys. 2020 May 4;21(7):107–18. doi: 10.1002/acm2.12895 (PMC7386195; doi:10.1002/acm2.12895)
Supplement: Supplementary file 1 — Fig S1 . Scree plot of the principal components for the HT treatment plans data. The eigenvalues give the variance explained by each PC. [file ACM2-21-107-s001.docx]

Figure S1 – Scree plot of the principal components for the HT treatment plans data. The eigenvalues give the variance explained by each PC.
