# Supplementary material for: On the complexity of helical tomotherapy treatment plans
Source: J Appl Clin Med Phys. 2020 May 4;21(7):107–18. doi: 10.1002/acm2.12895 (PMC7386195; doi:10.1002/acm2.12895)
Supplement: Supplementary file 2 — Fig S2 . TPS leaf open time histogram for three similar prostate cases. Plans were created to irradiate the prostate and seminal vesicles with 2 Gy per fraction. (a) MF of 2.445 and %LOT > pT‐20 ms = 3.2%; (b) MF of 2.007 and %LOT > pT‐20 ms = 11.4%; (c) MF of 1.809 and %LOT > pT‐20 ms = 23.0%. [file ACM2-21-107-s002.docx]

Figure S2 – TPS leaf open time histogram for three similar prostate cases. Plans were created to irradiate the prostate and seminal vesicles with 2 Gy per fraction. a) MF of 2.445 and %LOT > pT-20 ms = 3.2%; b) MF of 2.007 and %LOT > pT-20 ms = 11.4%; c) MF of 1.809 and %LOT > pT-20 ms = 23.0%.
