# Supplementary material for: On the complexity of helical tomotherapy treatment plans
Source: J Appl Clin Med Phys. 2020 May 4;21(7):107–18. doi: 10.1002/acm2.12895 (PMC7386195; doi:10.1002/acm2.12895)
Supplement: Supplementary file 3 — Table S1 . Spearman’s correlation coefficients, rs, and corresponding p‐values (within brackets) between 3D gamma passing rates with various criteria and the complexity metrics/nPCS for the head and neck and prostate plans. [file ACM2-21-107-s003.docx]

Table S1 – Spearman’s correlation coefficients, r_s_, and corresponding p-values (within brackets) between 3D gamma passing rates with various criteria and the complexity metrics/nPCS for the head and neck and prostate plans.

|  | **Head and Neck** | | **Prostate** | |
| --- | --- | --- | --- | --- |
|  | 3%/3 mm 10%TH | 3%/2 mm 10%TH | 3%/3 mm 10%TH | 3%/2 mm 10%TH |
| Modulation Factor (MF) | 0.110 (0.263) | 0.070 (0.481) | -0.159 (0.265) | -0.189 (0.184) |
| TT/Gy | 0.221 (0.023) | 0.249 (0.010) | -0.367 (0.008) | -0.332 (0.017) |
| Mean LOT | -0.263 (0.007) | -0.264 (0.006) | 0.119 (0.406) | 0.100 (0.486) |
| %LOT < 100 ms | -0.202 (0.039) | -0.201 (0.040) | -0.332 (0.017) | -0.260 (0.066) |
| %LOT < 50 ms | -0.292 (0.003) | -0.287 (0.003) | -0.222 (0.117) | -0.162 (0.255) |
| %LOT < 30 ms | -0.291 (0.003) | -0.300 (0.002) | -0.127 (0.373) | -0.068 (0.636) |
| %LOT > pT-20 ms | -0.285 (0.003) | -0.256 (0.008) | -0.194 (0.173) | -0.132 (0.356) |
| LOTV | 0.351 (0.000) | 0.339 (0.000) | 0.315 (0.000) | 0.357 (0.001) |
| PSTV | -0.349 (0.000) | -0.338 (0.000) | -0.326 (0.020) | -0.248 (0.080) |
| Modulation Index (MI) | -0.141 (0.150) | -0.148 (0.132) | 0.029 (0.842) | 0.047 (0.746) |
| CLS | 0.144 (0.143) | 0.160 (0.103) | -0.291 (0.038) | -0.266 (0.059) |
| CLS_in_ | -0.109 (0.270) | -0.140 (0.156) | -0.399 (0.004) | -0.325 (0.020) |
| L0NS | -0.048 (0.624) | -0.025 (0.799) | -0.357 (0.001) | -0.383 (0.006) |
| L1NS | -0.038 (0.703) | -0.039 (0.694) | -0.367 (0.008) | -0.318 (0.023) |
|  |  |  |  |  |
| nPCS | -0.325 (0.001) | -0.328 (0.001) | -0.369 (0.001) | -0.385 (0.005) |
