# Supplementary material for: On the complexity of helical tomotherapy treatment plans
Source: J Appl Clin Med Phys. 2020 May 4;21(7):107–18. doi: 10.1002/acm2.12895 (PMC7386195; doi:10.1002/acm2.12895)
Supplement: Supplementary file 4 — Table S2 . Spearman’s correlation coefficients, rs, and corresponding P (within brackets) TPS reported parameters and the pre‐treatment QA results for the head and neck, prostate and SRS plans. Values in bold correspond to significant moderate or strong correlations. [file ACM2-21-107-s004.docx]

Table S2 – Spearman’s correlation coefficients, r_s_, and corresponding p-values (within brackets) TPS reported parameters and the pre-treatment QA results for the head and neck, prostate and SRS plans. Values in bold correspond to significant moderate or strong correlations.

|  | **pitch** | **Gantry period** | **Gantry rotations** | **Couch travel** | **Couch speed** |
| --- | --- | --- | --- | --- | --- |
| **Head and Neck** |  |  |  |  |  |
| 3D global gamma analysis |  |  |  |  |  |
| 3%/3 mm 10%TH | -0.136 (0.165) | -0.101 (0.303) | 0.322 (0.001) | -0.294 (0.002) | -0.077 (0.433) |
| 3%/2 mm 10%TH | -0.126 (0.200) | -0.137 (0.165) | 0.377 (0.000) | 0.373 (0.000) | -0.035 (0.725) |
|  |  |  |  |  |  |
| **Prostate** |  |  |  |  |  |
| 3D global gamma analysis |  |  |  |  |  |
| 3%/3 mm 10%TH | 0.199 (0.160) | -0.057 (0.690) | -0.106 (0.458) | 0.178 (0.210) | 0.312 (0.026) |
| 3%/2 mm 10%TH | 0.175 (0.220) | -0.100 (0.483) | -0.057 (0.692) | 0.209 (0.141) | 0.343 (0.014) |
|  |  |  |  |  |  |
| **Stereotactic brain** |  |  |  |  |  |
| 3D global gamma analysis |  |  |  |  |  |
| 3%/2 mm 10%TH | - | 0.079 (0.580) | -0.342 (0.013) | -0.347 (0.012) | -0.079 (0.580) |
| 2%/2 mm 10%TH | - | **0.410 (0.003)** | **-0.657 (0.000)** | **-0.660 (0.000)** | **-0.410 (0.003)** |
| Film 3%/2 mm | - | 0.068 (0.632) | -0.182 (0.196) | -0.186 (0.187) | -0.068 (0.632) |
| IC %diff | - | 0.397 (0.006) | **-0.489 (0.001)** | **-0.486 (0.001)** | -0.397 (0.006) |
